# The effectiveness of enteral nutrition for patients with primary liver cancer: A randomized controlled study protocol

**DOI:** 10.1097/MD.0000000000023973

**Published:** 2021-01-22

**Authors:** Li Wang, Xiaoyue Wang, Xuejun Wang

**Affiliations:** Department of general surgery, Chun’ an County First People's Hospital, Zhejiang, China.

**Keywords:** enteral nutrition, hepatectomy, primary liver cancer, protocol

## Abstract

**Objective:**

The objective of this study is to explore the influence of the enteral nutrition on primary liver cancer patients after receiving hepatectomy.

**Method:**

This is a prospective randomized controlled research, which will be conducted between April 2021 and April 2022. Approval is obtained from the Research Ethics Committee of Chun’ an County First People's Hospital (A20201108). Patients who meet the following conditions will be included in this experiment:

(1) the patients aged 18 to 70 years;
(2) in line with clinical diagnostic criteria for primary liver cancer;
(3) planned liver resection for primary liver cancer;
(4) liver function status of Child-Pugh A.

Patients with the following characteristics are excluded:

(1) a history of other malignancy;
(2) mental disorder;
(3) severe diabetes or poor glycemic control;
(4) serious complications: bleeding and bile leakage;
(5) poor medical condition: renal failure, respiratory or heart failure.

Our investigation includes sixty patients who meet our inclusion criteria. The primary endpoints are length of postoperative hospital stay and liver function index. The secondary results involve the first flatus time and the first defecation time.

**Results:**

Table 1 indicates the postoperative outcomes between treatment group and control group.

**Conclusion:**

Enteral nutrition can improve recovery in the primary liver cancer patients after receiving hepatectomy.

**Trial registration:**

The protocol has been registered in Research Registry (researchregistry6275)

## 1 Introduction

Primary liver cancer is the seventh most prevalent cancer worldwide and the fourth leading cause of cancer death.^[1,2]^ The global death caused by the primary liver cancer is estimated at 780 thousand.^[3]^ The incidence rates in many countries have been rising and are expected to continue to rise in the next decade. For the primary liver cancer, its 2

main histologic types are intrahepatic cholangiocarcinoma (ICC) and hepatocellular carcinoma (HCC).^[4]^ HCC originates from the hepatocytes, with the most familiar background being inflammation, oxidative stress, and the potential liver disease, whereas ICC originates from the cholangiocytes around intrahepatic bile duct.^[5]^ Globally, HCC accounts for about 75 percent of all the liver cancers, and ICC accounts for about 12% to 15%.^[6]^ With the development of surgery, local treatment and systemic therapy, more and more patients are eligible to receive treatment that evidently improves the overall survival rate.^[7–9]^ Nevertheless, malnutrition is prevalent in the malignant liver disease patients. Optimization of the nutritional status can improve the liver function, and the nutritional status before operation is 1 of the keys to successful liver resection. In recent years, a variety of approaches have been proposed and utilized to keep the liver function and to facilitate the liver regeneration after hepatectomy. These involve the systemic interventions, for instance antibiotics during the perioperative period, and approaches to improve personal immunity and general health, for instance, probiotics.^[10,11]^ Among them, nursing care with nutritional support is also a vital approach to protect liver function. Studies have indicated that good nutritional status before operation can decrease the postoperative mortality, thus reducing the postoperative care costs. Currently, the effect of enteral nutrition in in patients undergoing hepatectomy are unclear. Therefore, we conduct this randomized control study protocol to explore the influence of the enteral nutrition on primary liver cancer patients after receiving hepatectomy.

## 2 Methods

This is a prospective randomized controlled research, which will be conducted between April 2021 and April 2022. It was registered in the in research registry (researchregistry 6275). Approval is obtained from the Research Ethics Committee of Chun’ an County First People's Hospital (A20201108).

### 2.1 Inclusion criteria and exclusion criteria

Patients who meet the following conditions will be included in this experiment:

(1) the patients aged 18 to 70 years;
(2) in line with clinical diagnostic criteria for primary liver cancer;
(3) planned liver resection for primary liver cancer;
(4) liver function status of Child-Pugh A.

Patients with the following characteristics are excluded:

(1) a history of other malignancy;
(2) mental disorder;
(3) severe diabetes or poor glycemic control;
(4) serious complications: bleeding and bile leakage;
(5) poor medical condition: renal failure, respiratory or heart failure.

Our investigation includes sixty patients who meet our inclusion criteria. All participants are randomly divided into the control group and treatment group. According to the randomization ratio of 1:1, there are 30 members in each group. The allocation is carried out as a block randomization, researchers and participants are blinded from the allocation sequence, which is saved in sequentially numbered, opaque sealed envelopes.

### 2.2 Study intervention

Treatment group: enteral nutrition (enteral nutritional suspension). This is a type of protein-enriched enteral nutrition (bottled preparations, net content of 1 500 mL bottle: 25 g of protein, 16.7 g of fat, 63.0 g of carbohydrates). This treatment (500–1000 mL) is given in addition to the regular diet orally in the 3 preoperative days, and administered in the 7 postoperative days orally or via a nasointestinal tube.

Control group: regular food intake. The requirement of target energy is set at 20 to 30 Kcal/kg per day. The total energy intake per day is identical to that in the treatment group. Before surgery, patients are fed on a low-fat diet. During the week after surgery, they are given liquid diet and semiliquid diet with the regular protocol.

Treatment compliance is rigorously assessed

(1) before surgery by checking the diary card
(2) within 7 postoperative days by checking nursing files.

The energy intake of every food is calculated according to Food Exchange List. Patients are asked to complete accurately in the diary card in order to conform to the protocol.

### 2.3 Outcome measures

The primary endpoints are length of postoperative hospital stay and liver function index. The secondary results involve the first flatus time and the first defecation time.

### 2.4 Statistical analysis

The original data are entered into the Microsoft Excel Spreadsheet 91 and they are analyzed through applying the software of Statistical Package for the Social Sciences (SPSS Inc., version 22.0, Chicago, IL). Afterwards, all the data acquired are represented through the appropriate characteristics, for example, standard deviation, and mean, median as well as percentage. And independent *t*-tests and *χ*2-tests are respectively utilized to analyze the categorical variable and continuous variable. *P* value less than .05 indicates that there is statistical significance.

## 3 Results

Table 1 indicates the postoperative outcomes between treatment group and control group.

**Table 1:** Postoperative outcomes between treatment group and control group.

Variables	Study group (N = 30)	Control group (N = 30)	*P* value
Length of postoperative hospital stay			
Time to first defecation			
Time to first flatus			
Alanine aminotransferase			
Aspartate aminotransferase			
Alkaline phosphatase			
Albumin			
Serum creatinine			
Alkaline phosphatase			
Serum alpha-fetoprotein			
Carcinoembryonic antigen			

## 4 Discussion

Liver cancer is one of the world's most familiar life-threatening diseases.^[12]^ Approximately 70% to 85% of primary liver cancer cases are HCC.^[13]^ Although progress has been made in the treatment of liver cancer, it is still one of the most difficult cancers to treat. For the early HCC patients, liver transplantation, local destructive treatments and surgery offer therapeutic potential. Nevertheless, HCC recurrence is still the main post-treatment problem, with a 5-year incidence rate of over 70 percent.^[14]^ Malnutrition is extensively believed to play a significant role in cancer patients and surgical patients.^[15]^ A lot of work has entered into the determination of the most proper role of the nutritional support in cancer patients during the perioperative period, and often comes to disappointing or vague conclusions.^[16]^ Although the parenteral nutrition has been utilized to prevent malnutrition from worsening in postoperative stage, it may promote inflammation and lead to severe liver complications. Enteral nutrition refers to the nutrition offered via the gastrointestinal tract which transports nutrients distal to oral cavity through stoma, catheter or tube.^[17,18]^ The role of enteral nutrition in liver surgery is still controversial. This is the first randomized study protocol to explore the influence of the enteral nutrition on primary liver cancer patients after receiving hepatectomy.

## 5 Conclusion

Enteral nutrition can improve recovery in the primary liver cancer patients after receiving hepatectomy.

## Author contributions

Xuejun Wang designs the protocol. Xiaoyue Wang reviews the protocol and performs the data collection. Li Wang finishes the manuscript. All of the authors approved the submission.

**Conceptualization:** Xiaoyue Wang.

**Data curation:** Xiaoyue Wang.

**Funding acquisition:** Xuejun Wang.

**Writing – original draft:** Li Wang.
